# The building blocks of successful translation of proteomics to the clinic

**DOI:** 10.1016/j.copbio.2017.12.011

**Published:** 2018-02-07

**Authors:** Paul Kearney, J Jay Boniface, Nathan D Price, Leroy Hood

**Affiliations:** 1 Integrated Diagnostics, Seattle, WA, United States; 2 Sera Prognostics, Salt Lake City, UT, United States; 3 Institute for Systems Biology, Seattle, WA, United States

## Abstract

Recently, the first two multiplexed tests using selective reaction monitoring (SRM-MS) mass spectrometry have entered clinical practice. Despite different areas of indication, risk stratification in lung cancer and preterm birth, they share multiple steps in their development strategies. Here we review these strategies and their implications for successful translation of biomarkers to clinical practice. We believe that the identification of blood protein panels for the identification of disease phenotypes is now a reproducible and standard (albeit complex) process.

## Introduction

The test development path from discovery to clinical practice is long, complex and challenging [1^••^]). Critical requirements include employing a systems-driven strategy to identify the candidate biomarkers; establishing clinical and analytical validation as well as clinical utility; developing a solid health economics basis for the test; gaining reimbursement; achieving intellectual property coverage; being in regulatory compliance with multiple agencies; gaining acceptance by practitioners and eventual inclusion in the appropriate medical guidelines; and obtaining funding to do all the above. Blood biomarkers will play a critical role in personalized and precision medicine to improve healthcare by distinguishing normal from diseased individuals, stratifying patients into drug responders and non-responders; stratifying diseases into distinct subtypes and developing biomarkers that are prognostic of disease outcomes.

While single analyte tests are currently more prevalent, clinical practice now includes multiplexed genomic tests (e.g. Oncotype DX [2]) and proteomic tests (e.g. Vectra DA [3]), the latter relying predominately on immunoassay platforms. The multiplexed panels of proteins make sense by virtue of the fact that in disease multiple biological networks become disease perturbed [4,5] and hence relevant proteins from these networks can contribute to the power of the diagnostic determinations. Multiplexed immunoassay test development is challenged by technological limitations, including availability of reagents, interference, cross-reactivity and lack of specificity [6]). Cross-reactivity is an enormous challenge, especially in complex mixtures of analytes such as blood. In contrast, SRM-MS has been proposed as a technology platform that can overcome many of these challenges, primarily due to its high specificity, high multiplexing capabilities and low assay development costs [7]. Nevertheless, the appearance of diagnostic tests based on SRM-MS technology in the clinic has lagged. The first two multiplexed SRM-MS diagnostic tests used in clinical practice are Xpresys^®^ Lung [8^•^,^9^] and PreTRM^®^ [10^•^,^11^], launched in 2013 and 2015, respectively.

Xpresys Lung is a blood test for assessing the cancer risk of lung nodules discovered by radiology such as CT scans. The national program for annual CT screening for lung nodules in high-risk individuals was initiated in 2015 largely based on the National Lung Screening Trial [12]. Each year approximately 1.6 million lung nodules are detected in the US alone [13] and that is expected to increase strikingly as the national screening program is fully implemented. The majority of these nodules are benign with an estimated 15–25% being malignant. Lack of precise diagnostics, however, results in 35–42% [14,15]) of benign nodules being over-treated with invasive procedures such as biopsies and surgeries. Xpresys Lung measures the relative expression of eleven proteins by SRM-MS, five being diagnostic and six used for normalization of signal, and uses this information to generate a probability estimate that a lung nodule is benign, providing molecular evidence for whether or not invasive procedures can be avoidable. Interestingly all five of these proteins are expressed in disease-perturbed networks found in lung cancer. A detailed analysis of the economic impact of the Xpresys Lung test is that it has the potential to save the American healthcare systems multiple billions of dollars a year by avoiding unnecessary procedures and surgeries.

PreTRM is a blood test that reports an individualized risk of spontaneous preterm birth (sPTB) in asymptomatic women in the middle of pregnancy (at 19–20 weeks) [10]. PTB is a leading cause of infant mortality and morbidity worldwide. In the U.S. it is the leading cause of neonatal death and death in children before age 5 years and the health-economic impact was estimated by the National Academy of Medicine (formerly Institute of Medicine) in 2005 to be in excess of $26 billion per year [16]. Prior to the development of PreTRM, intense research into the development of predictive algorithms based on clinical and demographic factors or using measured serum or vaginal biomarkers did not result in clinically useful tests. PreTRM measures the relative level of two proteins by SRM-MS. These measurements are combined into a risk estimate that a pregnancy will end in sPTB. High-risk pregnancies can then be treated with interventions such as progesterone and/or high intensity case management.

In comparing the development paths for Xpresys Lung and PreTRM we identify six shared strategies that contributed to their successful translation into clinical use.

- Adoption of systems-biology techniques for enhancing the likelihood of success in the selection of a large multiplexed panel of potential biomarkers (hundreds).
- Adherence to the National Academy of Medicine best practices [1^••^] for test development to mitigate risks such as overfitting as well as applying analyses to multiple genetically distinct populations to correct for the noise arising from human polymorphisms.
- Early attention to analytical performance in test development to ensure the test will be robust over time and reproducible over hundreds of thousands of tests to be performed.
- Avoidance of ‘loss in translation’, an inherent risk in translating a test from a discovery-grade technology platform to a commercial-grade technology platform.
- Utilization of expression correlation techniques for efficient large-scale SRM-MS assay development.
- Normalization techniques that address various sources of analytic and pre-analytic variation.

In what follows we will elaborate on the six strategies shared in the successful development and translation of Xpresys Lung and PreTRM to the clinic.

## Systems biology approach to selecting the initial candidates

Although SRM-MS technology allows for high proteomic multiplexing, it does not reach the comprehensive coverage of genomic technologies. Therefore a set of initial candidate proteins must be selected. For example, discovery stage SRM-MS assays for the Xpresys Lung and PreTRM tests began with 371 proteins and 242 proteins, respectively. The implication is that discovery stage SRM-MS assays must be designed to span specific subproteomes that hold the most promise for diagnostic discovery, such as for lung cancer or pre-term birth proteomes. This focus can be facilitated by systems-biology techniques that harness multiple lines of evidence, including relevant existing high-throughput data analyses and sub-proteome sets that span relevant disease-perturbed networks and that are likely to be reliably detectable in the blood.

For Xpresys Lung, proteins were identified that were likely to be blood-based biomarkers of lung cancer. Literature searches and empirical studies were designed to identify cell-surface (membrane proteins are often cleaved and released into the blood) and secreted proteins differentially expressed by lung cancer cells [8^•^]. These proteins were then filtered using public resources such as the Peptide Atlas or previous detection in blood. Finally, proteins were prioritized for inclusion on the discovery assay based on specificity to lung tissue.

It turned out that 190/371 discovery proteins could be routinely detected in the blood. These 190 proteins were scored according to their ability to distinguish between samples of individuals with 72 benign and 72 malignant tumors. Thirty-two of these proteins performed significantly better than the others.

For PreTRM, protein biomarker candidates were selected by likelihood of being detectable in blood and for their presence in molecular networks implicated in pregnancy complications. Annotation as either cell-surface or secreted proteins guided selection from pregnancy relevant literature searches, and preliminary *de novo* serum proteomic discovery studies, by both mass spectrometry and an immunoassay panel screen (rules-based medicine), focused on evidence of dysregulation in preterm birth and preeclampsia. Protein candidate filtering and surrogate proteotypic peptide selection utilized public (protein/peptide atlas) and private databases, and full-scan MS/MS data from shotgun proteomic studies [10^•^].

A second application of a systems approach is the identification and prioritization of “cooperative” proteins. Whereas discovery-stage studies often rank analytes by univariate methods, as described above, cooperative proteins are ranked based on performance on protein panels (i.e. the best “team players”) rather than on individual performance. This strategy is motivated by the intent to capture the integrated behavior of proteins within disease-perturbed networks.

In the case of Xpresys Lung, computational methods were utilized to identify cooperative proteins that appeared most frequently on the best performing protein panels by sampling the combinatorial possibilities. This was executed by creating a million panels of 10 from the 32 most effective proteins from the univariate analyses. We then used computational methods to identify the most cooperative proteins — and 5 of the 32 fell into that category and these were the diagnostic proteins included in the Xpresys Lung test. These 5 proteins were then validated against the plasmas of 52 individuals with benign and 52 individuals with malignant tumors. The important point is that proteins included in the Xpresys assay may not have the best individual performance but did appear frequently on winning “teams”. PreTRM used a similar strategy where ratios of pairs of proteins were assessed for best predictive performance. “Reversals” were formed from the ratio of up-regulated proteins over down-regulated proteins to find the best predictive pair and the best gestational age for prediction [8^•^,^9^,10^•^].

**Figure 1:**
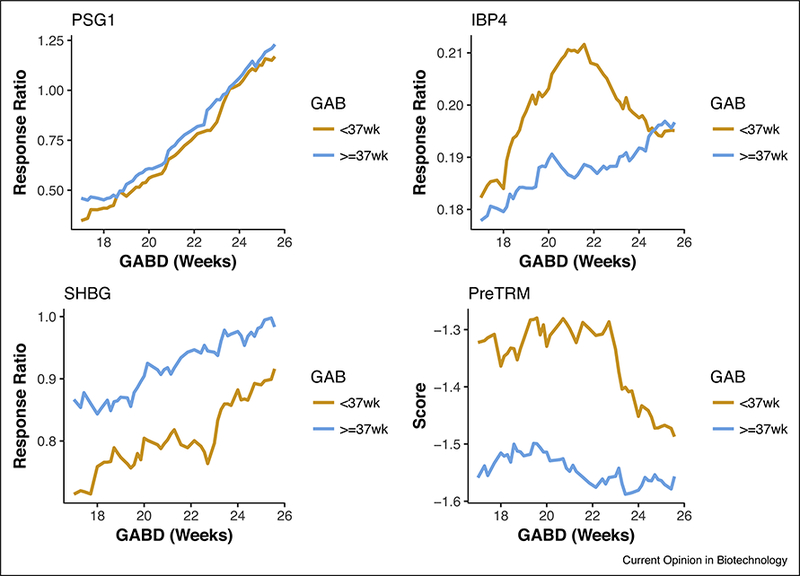
Analyte kinetics.

A third application of systems biology is in the design of the discovery studies. Not surprisingly, the pregnancy proteome changes rapidly over gestation, with biomarkers becoming relevant only at certain times during pregnancy [17]. Viewing gestation as a dynamic system over time, discovery studies for PreTRM surveyed serum drawn broadly in gestation (17–28 weeks) to identify the dependency of protein levels and diagnostic signal on gestational age (Figure 1).

Similarly, in the context of lung cancer, the size of the lung nodule is correlated to cancer stage and cancer biology. For this reason, the discovery study for Xpresys Lung spanned a wide range of lung nodule sizes (8–30 mm in diameter) to assess the sensitivity of the proteins assays to changes in nodule size. Some proteins have sustained differential expression levels across broad changes in nodule size, while others do not.

## Adherence to National Academy of Medicine guidelines for best practices

Another major factor in the design of both successful clinical tests was adherence to the guidelines set out by the National Academy of Medicine’s report on development of omics-based tests for clinical trials [1^••^]. One of the major considerations in the development of an omics-based test is the possibility of overfitting to the data, resulting in often-dramatic underperformance of tests when they move into independent validation in different test populations [18]. Thus, the study design must mitigate these risks and avoid the issue of many candidate biomarkers not holding up through the long road to clinical use. Considerations include testing the biomarkers in the discovery stage in multiple different populations (thus averaging out the varied genetic and environmental influences), as well as setting up a discovery and test validation stage for the development of the test. The test must be locked down (i.e. be completely specified before moving into the test set and then remain unaltered), including the laboratory process, the analytes that are measured and, the accompanying computational algorithm applied to the measurements (the log regression approach for Xpresys Lung and the protein reversal approach for PreTRM). Independent validation must then be conducted, not just on completely new samples, but also include samples from new sources/sites. This ensures that the performance in validation best estimates the performance of the test for clinical use. All of these steps and the other guidelines of the National Academy of Medicine report were critical to the successful launch of both the Xpresys Lung and PreTRM biomarker panels.

A second major recommendation from the guidelines was to use geographically discrete populations for discovery and validation of blood biomarker tests so as to avoid the noise that comes from human genetic polymorphisms. For example, in the Xpresys Lung test we carried out the discovery stage with plasmas from 4 different populations (72 benign and 72 malignant nodules) and with the validation stage we analyzed new samples from the original 4 populations and added samples from a fifth population (52 benign and 52 malignant nodules).

## Assessing analytical performance early in development

In the translation of a test to the clinic, consideration of analytical performance early in development is of paramount importance. The reason is simple: a test successfully translated to the clinic may need to be performed reproducibly on hundreds of thousands of samples, year after year.

Consider the sample workflow used in both the Xpresys Lung and PreTRM tests: first, sample acquisition and shipping, second, depletion of high abundance proteins, third, tryptic digestion of proteins into peptides and then fourth, analysis by liquid chromatography (LC)-SRM-MS. Each of these steps introduces analytical variation. Analytical variation can amplify significantly when one considers that the process involves different phlebotomists, depletion columns, reagents, mass spectrometers and operators, among other variables. Analytes measured in the Xpresys Lung and PreTRM tests have analytical variation over all these sources of variation in total under 12% [11]. This level of reproducibility requires significant quality control to achieve.

To achieve this precision, individual protein analytes were ranked by both their analytical robustness and diagnostic performance. Focused studies were designed and executed to identify proteins robust to variation in sample acquisition, column depletion, trypsin digestion and LC-MRM-MS analysis. Some proteins are simply more robust to these sources of analytical variation than others, making them more suitable for reproducible use in clinic diagnostics.

Diagnostic tests are performed on freshly acquired samples. However, it is frequently the case that discovery, clinical validation and clinical utility studies are performed on samples from archival biobanks of samples that were acquired using different acquisition protocols and stored for varying lengths of time. Xpresys Lung and PreTRM analytes were assessed for robustness to storage conditions and storage time. If a test is not *designed* to work on both prospective and archival samples, then this limits studies to a prospective design, which increases development costs and timelines dramatically. Furthermore, prior to clinical use of the test on freshly acquired (non-archived) samples, rigorous bridging studies must be performed to demonstrate equivalent test performance between the initial discovery conditions and the conditions of clinical application. Both Xpresys Lung and PreTRM are robust, by design, to a broad range of analytical variation including storage acquisition and storage conditions. This allows for their applicability to both retrospective and prospective study designs. We believe this should be a critical feature of new blood protein biomarker panels — for it increases enormously the possibilities for discovery.

## Avoidance of ‘loss in translation’ across technology platforms

Typically, diagnostic tests are developed on technology platform A and then deployed for clinical use on a different technology platform B. The reason for this is that technology platform A is suitable for low throughput, higher cost, highly multiplexed discovery studies whereas technology platform B is suitable for high throughput, lower cost and lower complexity clinical testing. An example is discovery or verification using a SRM-MS platform with deployment using a platform based on capture agents such as antibodies or (modified) aptamers. These platforms can be limited by cross-reactivities, especially in complex mixtures such as blood — hence they typically work better clinically with panels with just a few proteins (the cost of optimizing each antibody pair for ELISA assays is significant — and even with optimization significant cross-reactivities may remain. Emerging technologies for protein capture agents hold great promise to meet these challenges and reduce cross-reactivities [19,20].

However, the translation from technology platform A to B carries significant risk. First, the analytical performance of analytes measured on platform A may differ significantly from analytes measured on platform B. Second, there is no guarantee that the analytes measured on technology platform A are the same analytes as measured by technology platform B, and thus diagnostic performance may deteriorate substantially. Furthermore, the translation from technology platform A to B can come at significant cost and time. For example, the translation from a SRM-MS assay to an antibody assay may take 12–24 months of additional time to validate and significant resources, if it validates at all.

‘Loss in translation’ is the acknowledgement of the risks and costs associated with deployment on a platform that differs from discovery. Xpresys Lung and PreTRM were intentionally developed and deployed using the same technology platform for discovery and clinical analyses to eliminate this risk and shorten time to the clinic-a major benefit of using SRM-MS. The economics of the test to be developed plays an important role here. Using a SRM-MS platform for development and commercial deployment is feasible for high-value tests such as Xpresys Lung and PreTRM. However, for low-cost tests (e.g. single analyte assays), it may be more prudent to use a two-platform strategy.

## Efficient assay design

Ideally, the transitions (peptides) monitored by an SRM-MS assay truly derive from the proteins intended to be measured. The complication here is that the same peptide may be represented in many proteins. One approach to avoiding this ambiguity is to use the SRM Atlas that has validated peptide assays for most human proteins [21^••^]. We have also employed an approach to ensure fidelity by developing assays where, say, at least four peptides per protein are included in the assay. The problem with this approach, in the context of blood-based tests, is that usually only high abundance blood proteins admit such high peptide coverage, which severely limits the SRM-MS assay. This is a safe but unnecessarily conservative approach.

Another approach is to develop labeled peptides for every endogenous peptide in the assay, which can be used to confirm fidelity. However, for discovery assays of hundreds of proteins, this can be cost-prohibitive.

**Figure 2:**
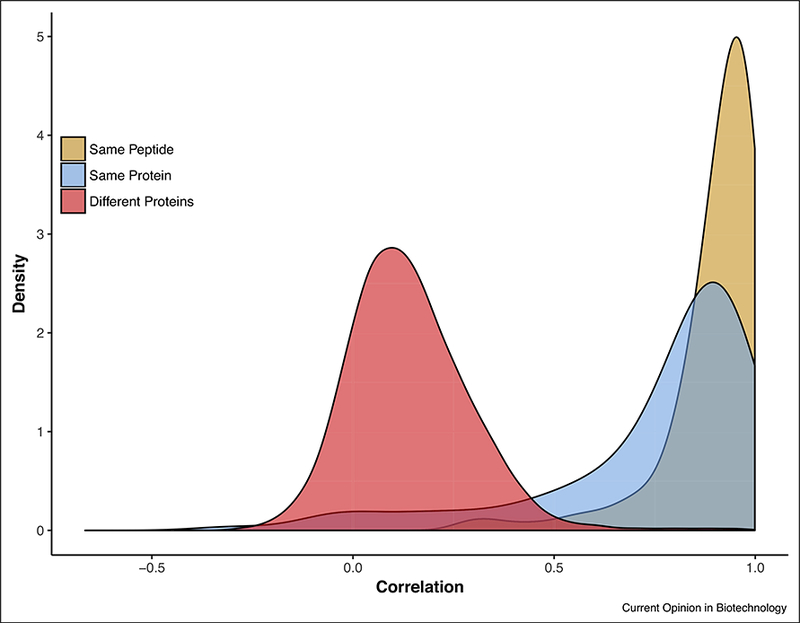
Transition correlation.

An alternative approach innovated in the development of the Xpresys Lung assay and also utilized in the PreTRM assay development is peptide correlation [22]. This approach trades off peptide redundancy in the assay for analyzing more clinical samples. The premise is straightforward: If two peptides, not necessarily unique, are derived from the same protein then their expression levels will be highly correlated across clinical samples assayed; conversely if they are not then they will not be correlated. This approach requires only two peptides per protein to generate a high fidelity SRM-MS assay, making the approach clinically relevant to a much broader set of proteins. Furthermore, the approach allows for a statistical treatment of the false discovery rate, an assessment of confidence, based on the correlation value using the empirical distribution of correlation scores for peptides from different proteins. This is illustrated in Figure 2 for the PreTRM discovery assay. In particular, it is highly unlikely (<2% probability) that two transitions with a Pearson correlation of 0.5 or higher are actually from different proteins.

## Normalization techniques to address analytic and pre-analytic variation

The foundation of normalization in Xpresys Lung and PreTRM is a ratio A/B of proteins A and B. Using protein ratios controls for both pre-analytical and analytical sources of variation as both proteins A and B undergo the same effects of pre-analytical and analytical variation in the same sample. There are examples in the literature of the usefulness of focusing on marker pairs as a basic diagnostic unit [23–25].

In the case of Xpresys Lung, normalization is performed by a set of six endogenous proteins combined with labeled-peptide normalization. For the proteins of Xpresys Lung, an analytical study varying all aspects of the proteomic workflow and spanning six months, the median technical coefficient of variation (CV) was 11.9%. Several proteins assessed in this study were detected at the low ng/ml concentration levels.

For PreTRM the normalization solution is even simpler with the classifier having the form IBP4/SHBG, where protein IBP4 is up-regulated and protein SHBG is down-regulated in pregnancies destined for premature delivery. This strategy has two advantages. First, it amplifies signal to noise issues. Second, it allows for normalization under the assumption that IBP4 and SHBG experience the same pre and post analytical effects in the same sample. The analytic variability measured for the ratio of IBP4/ SHBG is approximately 50% of the analytical variability of the two individual proteins.

## Discussion

We have reached the point where advancing a multiplex proteomic assay into the clinic, using systems strategies and multiplexed SRM-MS technology, can be carried out routinely, based on experience and hindsight. In this opinion piece we present the first two examples of developing protein panel tests for use clinically that are based on the systems approach/multiplexed SRM-MS technology. Importantly these two tests are in entirely different disease indication areas but share at least six common development strategies, which lends to the generalizability of these strategies. Ideally, translation to the clinic should build upon experience and become increasingly methodical and less experimental.

Multiple strategies are presented here: the intelligent construction of systems-based assays; adhering to best practices for test development and avoiding well-established pitfalls such as complex classifier models and overfitting; designing assays of high analytical robustness for routine use in the clinic; and assay development methods that save time, costs and are resource efficient. All of these strategies have in common the element of intentional design. To reach the clinic, an assay must be intentionally designed to circumvent each of these challenges. We hope this perspective helps clinical proteomics realize its diagnostic potential as a platform capable of highly multiplexed measures of a biomolecule class with close association to health and disease.

Selection of a technology platform that can comprehensively interrogate clinically meaningful biology is a requirement. Proteomic platforms are excellent candidates because protein expression is a very good direct surrogate of actual biological function in a manner that most nucleic acid tests are not. Going forward, these technologies can also be deployed in highly multi-omic settings for monitoring health [26]. Indeed, our feeling is that with clear clinical assays, it is now possible to generate blood protein biomarker panels for virtually any disease or phenotype change.
